# Individual-level variations in malaria susceptibility and acquisition of clinical protection

**DOI:** 10.12688/wellcomeopenres.16524.3

**Published:** 2022-02-25

**Authors:** John Joseph Valletta, John W.G. Addy, Adam J. Reid, Francis M. Ndungu, Yaw Bediako, Jedida Mwacharo, Khadija Said Mohammed, Jennifer Musyoki, Joyce Mwongeli Ngoi, Joshua Wambua, Edward Otieno, Matt Berriman, Philip Bejon, Kevin Marsh, Jean Langhorne, Chris I. Newbold, Mario Recker

**Affiliations:** 1 School of Mathematics and Statistics, University of St. Andrews, St. Andrews, UK; 2 Malaria Immunology Laboratory, Francis Crick Institute, London, UK; 3 Parasite Genomics, Wellcome Trust Sanger Institute, Hinxton, UK; 4 KEMRI/Wellcome Trust Research Programme, Kilifi, Kenya; 5 West African Centre for Cell Biology of Infectious Pathogens, University of Ghana, Accra, Ghana; 6 Centre for Tropical Medicine and Global Health, University of Oxford, Oxford, UK; 7 MRC Weatherall Institute of Molecular Medicine, University of Oxford, Oxford, UK; 8 Centre for Ecology and Conservation, University of Exeter, Penryn, UK

**Keywords:** Plasmodium falciparum, clinical malaria, malaria susceptibility, naturally acquired immunity, spatial heterogeneity, longitudinal cohort study

## Abstract

After decades of research, our understanding of when and why individuals infected with *Plasmodium falciparum* develop clinical malaria is still limited. Correlates of immune protection are often sought through prospective cohort studies, where measured host factors are correlated against the incidence of clinical disease over a set period of time. However, robustly inferring individual-level protection from these population-level findings has proved difficult due to small effect sizes and high levels of variance underlying such data. In order to better understand the nature of these inter-individual variations, we analysed the long-term malaria epidemiology of children ≤12 years old growing up under seasonal exposure to the parasite in the sub-location of Junju, Kenya. Despite the cohort’s limited geographic expanse (ca. 3km x 10km), our data reveal a high degree of spatial and temporal variability in malaria prevalence and incidence rates, causing individuals to experience varying levels of exposure to the parasite at different times during their life. Analysing individual-level infection histories further reveal an unexpectedly high variability in the rate at which children experience clinical malaria episodes. Besides exposure to the parasite, measured as disease prevalence in the surrounding area, we find that the birth time of year has an independent effect on the individual’s risk of experiencing a clinical episode. Furthermore, our analyses reveal that those children with a history of an above average number of episodes are more likely to experience further episodes during the upcoming transmission season. These findings are indicative of phenotypic differences in the rates by which children acquire clinical protection to malaria and offer important insights into the natural variability underlying malaria epidemiology.

## Introduction

Individuals growing up in *P. falciparum* malaria endemic areas acquire a general state of immunity against clinical malaria through repeated exposure to the parasite. This process of naturally acquired protection is still poorly understood ^1^ but believed to involve the build-up of a repertoire of immune responses against the myriad of antigenic targets that the parasite displays over its lifecycle during *in vivo* infections ^2– 4^. Although protection against life-threatening disease may be acquired through a small number of infections only ^5^, individuals remain prone to experience clinical episodes throughout childhood and sometimes even into their late teens or early adulthood, depending on the intensity of transmission (acquisition of protection is generally faster in high transmission settings with year-round transmission than in settings with little and interrupted transmission ^6^).

Research into the complexity of clinical protection often relies on cohort studies, where antigen-specific immune responses are correlated against the incidence of disease. These studies have been key in advancing our understanding of the role of variant surface antigens in both disease severity and natural acquired protection, for example, and are instrumental for the discovery of novel candidate targets for vaccine research ^7, 8^. Unfortunately, finding robust correlates of protection is made complicated by at least two factors. First, the observed effect sizes are often small, in particular with respect to the variability of the underlying data, and it is difficult to ascertain how much these reflect the true effects sizes due to the many confounding factors influencing the observations. Second, protection itself is not a dichotomous phenotype but rather related to an individual’s risk, or probability of developing disease if infected. The latter is particularly problematic because in many cases we simply do not know whether someone got infected during the study period or not.

In order to capture some of the spatial and temporal variation in disease transmission, and thus some of the uncertainty underlying the risk of an infection *per se*, Olotu *et al.* ^9^ previously proposed an exposure index, which provides a quantitative marker for the risk of infection experienced by an individual in space and time. What this and other research ^10^ found was that disease prevalence, or transmission intensity, is not necessarily homogeneous across space or time. In fact, even small geographic regions can exhibit malaria *hotspots*, where individuals have a notably higher chance of getting infected than in surrounding areas ^11– 13^. The temporal stability of these hotspots is variable, however. Whereas some hotspots can be persistent because of environmental or geographic factors, such as proximity to standing water and so to mosquito breeding sites, others might just persist for the duration of a transmission season or two. One way or the other, this heterogeneity in exposure must be considered as an important source of variability in an individual’s risk of experiencing a clinical episode in a given year, irrespective of their degree of protection.

Longitudinal cohort studies are ideally placed to offer more detailed insights into the process of naturally acquired protection (e.g. 14). That is, following individuals over time and recording their clinical episode histories can provide important information on how previous infections relate to a child’s future risk of disease and how this changes as they grow up under repeated exposure to the parasite. Here we made use of data generated from a long-term birth-cohort study in Kenya. Using individual-level episode histories together with spatio-temporal disease prevalence data we demonstrate that children exhibit a high degree of phenotypic variability regarding their development of protection from clinical malaria. We further identified independent risk factors for clinical disease, which should help to guide future studies aimed at finding robust immune signatures of anti-malarial protection.

## Methods

### Study population

The study was conducted through the KEMRI-Wellcome Trust Research Programme (KWTRP), Kilifi, Kenya. The children investigated were part of the long-term birth cohort in Junju. Recruitment into the cohort was voluntary following sensitisation of the whole area without specific spatial or demographic selection criteria, such thatthe cohort provides an unbiased spatial representation of the village as a whole. Children were recruited into the cohort at birth and actively monitored on a weekly basis for detection of malaria episodes until 15 years of age. See 15 for further information regarding the Junju cohort.

*P. falciparum* ( *Pf*) episodes, defined as a body temperature > 37.5°C and 2,500 parasites per microlitre of blood, are diagnosed during weekly active surveillance, where auxiliary body temperature and/or recent history of fever were recorded. Blood samples were taken from febrile children and *Pf* infection initially detected by rapid diagnostic test (RDT) and confirmed by microscopy. Apart from the children’s infection status and history of clinical malaria, we also had access to their date of birth, spatial location of their homestead, and sickle cell trait status (AA or AS; homozygous (SS) individuals were removed from this analysis).

### Ethical considerations

Approval for human participation in this cohort studies was given by Kenya Medical Research Institute Ethics Research Committee, and research was conducted according to the principles of the Declaration of Helsinki, which included the administration of informed consenting in the participant’s local language.

### Sample selection

Our statistical analyses are based on a subset of samples taken between 2006 and 2018 and include children between the ages of 1 and 12 years ( *n* = 544, total number of samples *N* = 3767). Unless stated otherwise, these samples include children with the known malaria protective sickle cell trait (heterozygous, AS; *n* = 92, total number of samples *N* = 618). Note, throughout the analysis we refer to *n* as the number of individuals, and refer to *N* as the number of samples (with *N > n*, as individuals were sampled longitudinally over the course of the study).

### Prevalence index

To capture and compare the spatial distribution of malaria over time within this cohort we devised a *prevalence index* ( *PI*), which provides a summary statistic of the annual prevalence at location *i* in year *t* by summing over all individuals *j* with recorded malaria episodes in that year in the neighbourhood of *i*, weighted by their spatial distances, *D _ij_*. It is given as

$$PI_{i,t}=\frac{\sum_{j}Z_{j,t}e^{-aD_{ij}}}{\sum_{j}e^{-aD_{ij}}}$$

where *Z _j,t_* is a binary operator indicating whether whether the individual had a recorded episode or not. Note, locations and individuals are used somewhat interchangeably, which also accounts for the occasions where multiple children from the same homestead are simultaneously enrolled. Although clustering at the individual household-level has previously been described (e.g. 16), we here did not consider such fine spatial resolution, especially as we only focused on children and no other family / household members.

The spatial weighting factor a determines how rapidly the influence of an infection at *j* on the prevalence at *j* declines with increased spatial distance. Note, this measure does not rely on a fixed spatial radius to determine the neighbourhood of *i*; instead, the chosen functional form of the spatial weighting means it naturally converges towards 0, such that far away locations have little to no effect. The value of *a* (here *a* = 2) has a significant influence on the spatial heterogeneity of *PI*, with smaller values leading to smaller differences in *PI* between distant locations, and vice versa. Initial attempts to estimate *a* form the data were not satisfactory due to large variations between estimates based on individual years. It was therefore decided to determine *a* by maximising the correlation between the resulting prevalence index and clinical episode for all individuals and all time points ( *PI* ~ *Z*).

### Statistical modelling

In order to determine potential risk factors underlying an individual’s probability of experiencing a clinical malaria episode, we built a Bayesian hierarchical logistic regression model. The modelled outcome was *GotEpisode*, a binary response variable (yes/no) indicating whether an individual experienced a clinical episode in a given year or not. The explanatory variables were *PI*, *Age*, *BirthQuarter*, and *Genotype* (AA or AS, heterozygous sickle cell trait). To test the effect of an individual’s previous episode history, we built an additional model using *PrevEpisodes* along-side *PI* and *Age*, and the interaction between *Age* and *PrevEpisodes*, as explanatory variables. Here, *PrevEpisodes* refers to the child’s number of previously recorded clinical episodes. In both cases we included a child-specific intercept (commonly known as a random effect in the frequentist literature), to accommodate the longitudinal nature of these data, where individuals were repeatedly sampled over multiple years over the duration of the study. To assess the effect of between-child variation, we compared the model against an equivalent non-hierarchical model assuming complete independence of all data points. Model comparison was done based on the models’ expected log predictive density (ELPD) using Pareto smoothed importance-sampling leave-one-out cross-validation (PSIS-LOO) from the *loo* package in R. All continuous variables were centred and scaled (i.e. set mean = 0 and standard deviation = 1) to improve model convergence and to allow for better comparison between the estimated effect sizes. A zero-mean normal prior (with standard deviation = 10) was placed on all regression coefficients, and a gamma prior (with shape and scale parameter = 1) was placed on the standard deviation for the group-level effect (i.e. the standard deviation for the child-specific intercepts). The model parameters were jointly inferred by means of MCMC sampling, using the *rstanarm* (version 2.21.1) R package. A total of 2000 iterations (including 1000 iterations for warm-up) were used for each of four chains run in parallel. Convergence was assessed via trace plots and the Rhat convergence diagnostic. Marginal effects for the explanatory variables on the response were plotted using the *marginal_effects* function in the *brms* (version 2.13.5) R package. R version 3.6.3 was used to produce all the figures and perform all of the analyses.

## Results

### Malaria prevalence fluctuates over time and space

**Figure 1.**
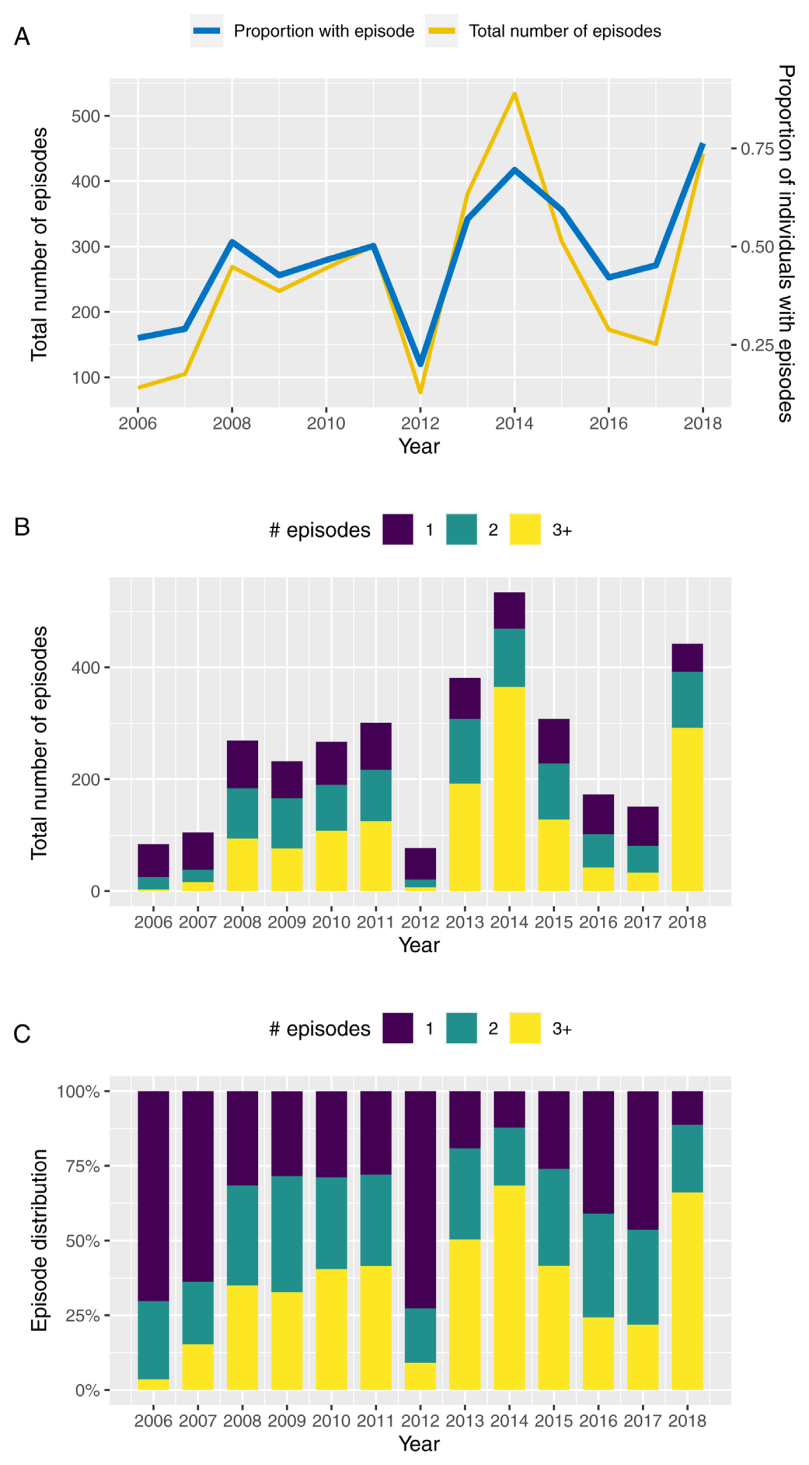
Temporal variation in malaria incidence. **A**. Temporal variance in the number of clinical episodes and proportion of individuals with a recorded episode in Junju, Kenya, between 2006 and 2018. **B**, **C**. Temporal variation in the distribution of children experiencing 1 (blue bars), 2 (green bars), or ≥ 3 clinical episodes (yellow bars) per year between 2006 and 2018, highlighting an *excess* of multiple episodes in years of high transmission (2014 and 2018).

Figure 1A illustrates the annual variation in the total number of clinical episodes and proportion of individuals with a recorded clinical episode between 2006 and 2018 based on children under the age of 12 years enrolled in this cohort. Although prevalence and total number of episodes follow a qualitatively similar trend, years of high prevalence, such as 2014 and 2018, are also characterised by individuals experiencing multiple episodes in the same year ( Figure 1B and 1C). That is, in those years we not only see more individuals with a clinical episode but a disproportional increase in the numbers of episodes per individual, which could be indicative of a prolonged or more intensive transmission season or a possibly a change in the circulating parasite population.

**Figure 2.**
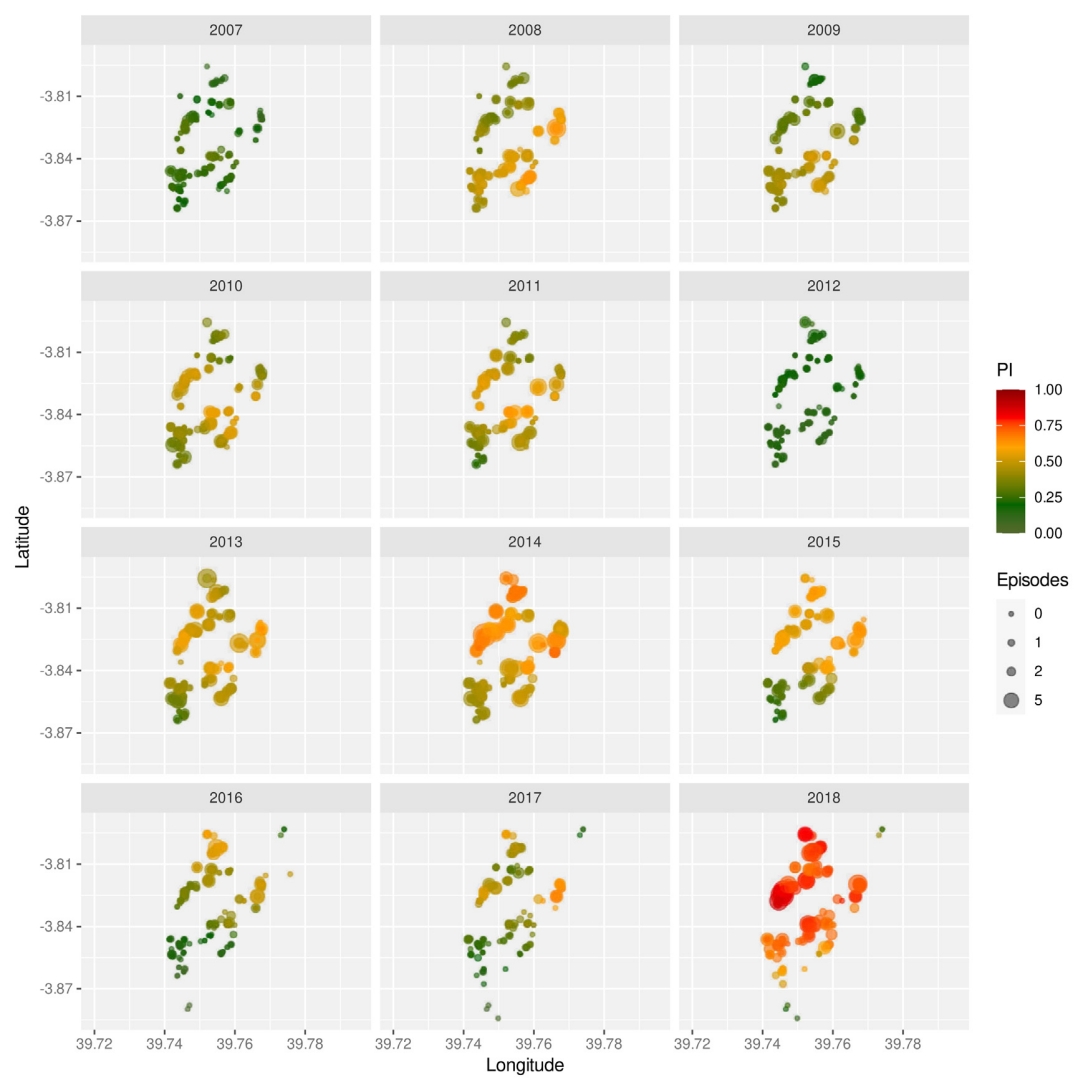
Spatio-temporal variation in malaria prevalence. Geographic homestead location of children enrolled in the Junju cohort, stratified by year. The size of each point correspond to the child’s number of recorded episodes that year, with the colour indicating their prevalence index ( *PI*).

The strong inter-annual variability is also mirrored in the spatial distribution of malaria prevalence, as shown by means of the prevalence index ( *PI*, see Methods) for the years 2007 to 2018 in Figure 2. Not only do these maps show significant year-on-year fluctuations but they also reveal temporal trends with some regions having much higher prevalence rates in some years than others. Furthermore, we find that in high prevalence years (2014 and 2018), malaria incidence was not confined to particular areas but was observed almost across the entire cohort, which could be indicative of more intense and/or longer transmission seasons. Note, because *PI* is based on recorded symptomatic episodes in children only we cannot say whether the observed spatial patterns are indicative of dynamically changing transmission hotspots or of potential heterogeneities in the immunity landscape across the cohort, or both.

### High individual-level variability in clinical episode histories

**Figure 3.**
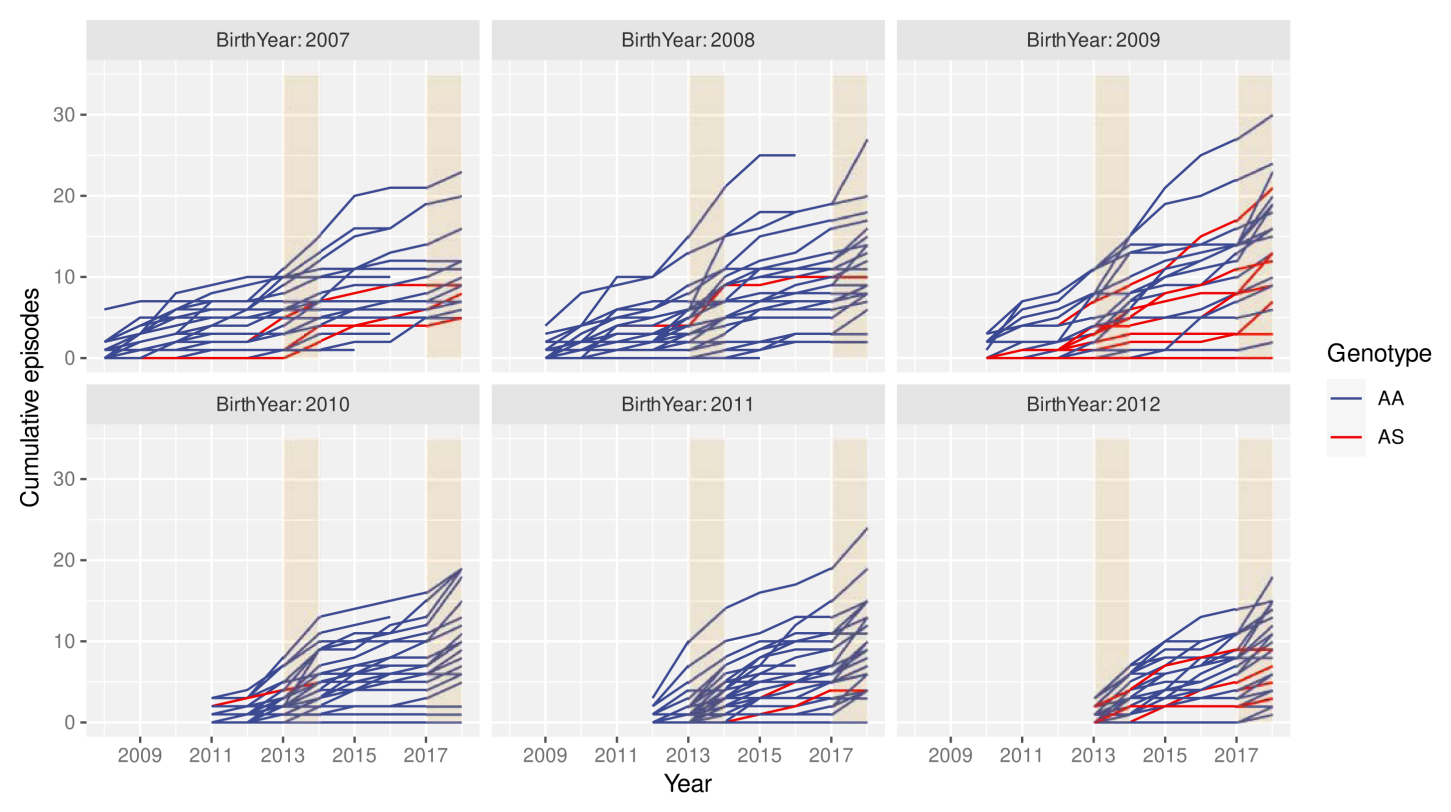
High variance in clinical episode histories. Graphs showing individual children’s cumulative number of clinical malaria episodes over time, stratified by birth year. Red lines indicate children with the sickle cell trait (AS). Orange rectangles highlight high malaria transmission years (2014 and 2018).

The spatio-temporal analyses above imply that individuals growing up in the cohort are likely to experience different levels of exposure to the parasite at different points during their lives. Consequently, individual histories of clinical malaria episodes are expected to be equally variable. This is exemplified in Figure 3, where individual episode histories, by means of the cumulative number of episodes, are plotted over time for children born between 2006 and 2011, stratified by the individuals’ year of birth and genotype (AA/AS). Besides the high variance in the rate at which individuals acquire clinical episodes, these graphs also suggest that neither the cumulative number of episodes nor the rate at which episodes are acquired (i.e. number of episodes per year) are simply a function of age. That is, in years of particularly high transmission, e.g. in 2014 or 2018, most children seem susceptible to a clinical episode regardless of their age or previous episode history.

### The effect of age on the risk of clinical malaria

**Figure 4.**
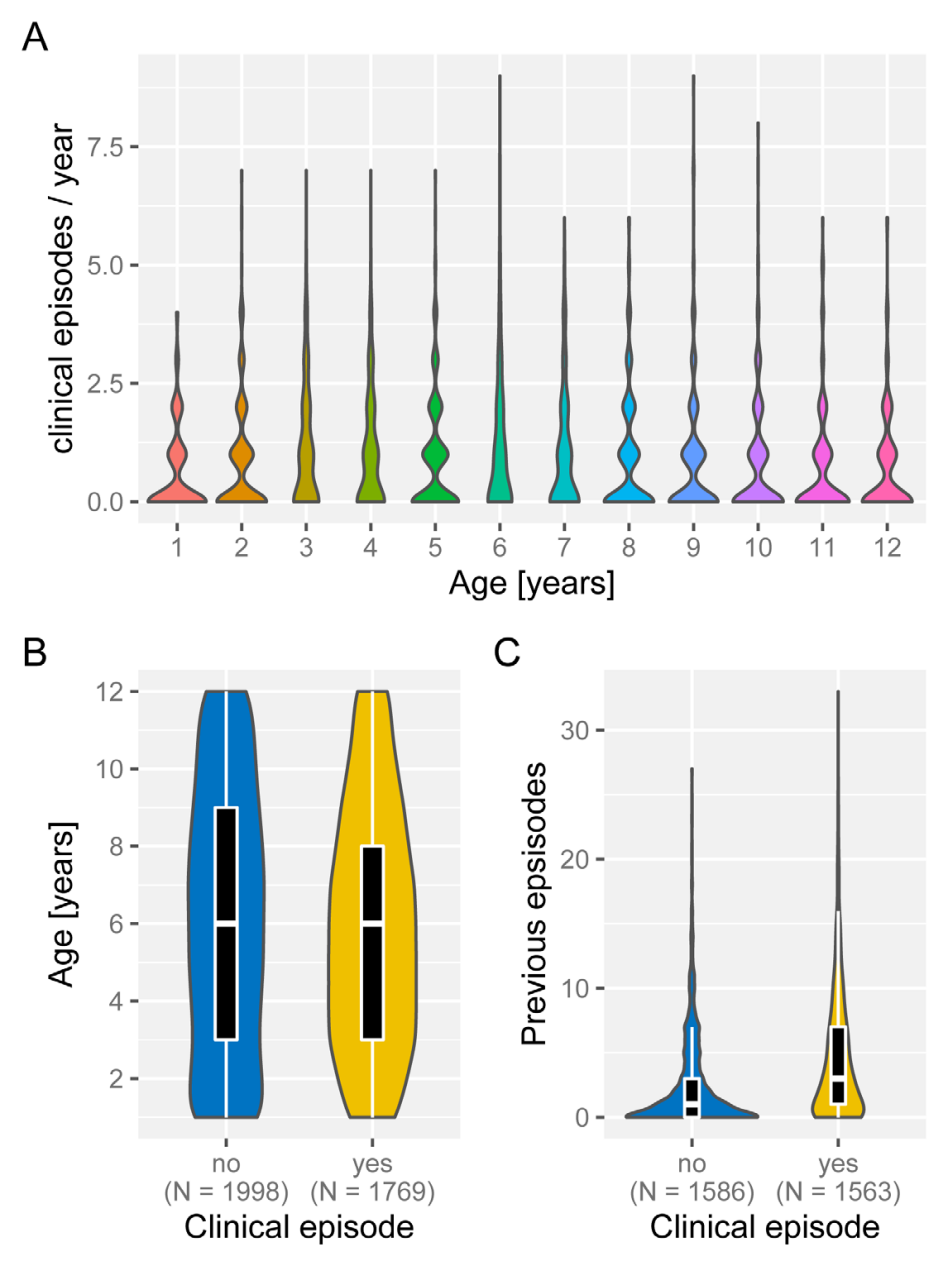
Effect of age and exposure history on risk of clinical malaria. Box and whisker plots indicate the median and inter quartile ranges of age and previous number of episodes of individuals with or without a recorded episode in a given year. Due to confounding effect of the sickle cell trait, AS individuals were removed in ( **C**).

**Figure 5.**
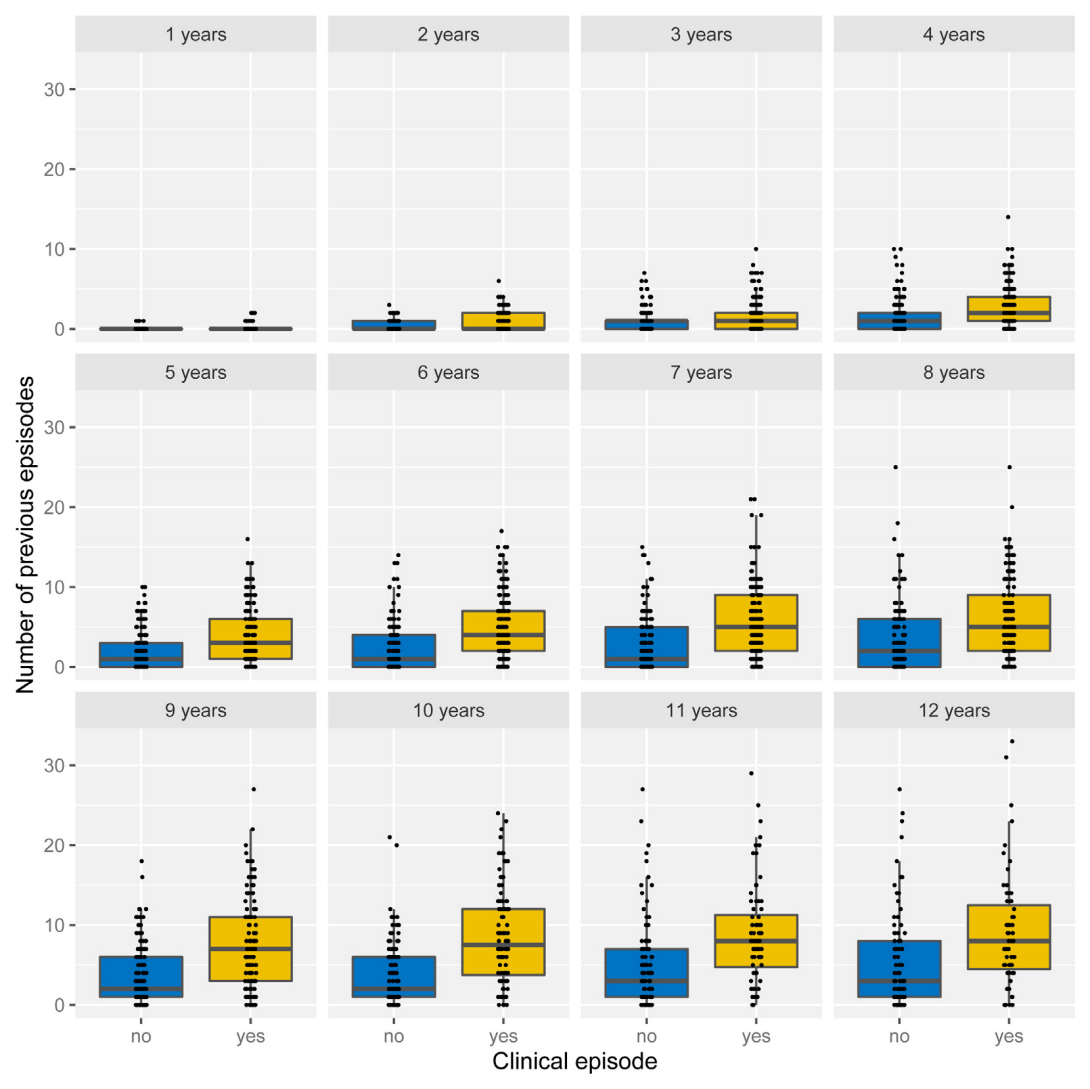
Age-stratified distribution of episode history in relation to risk of clinical malaria. Box and whisker plots indicate the median and inter quartile ranges of age and previous number of episodes of individuals with or without a recorded episode in a given year. Individuals with sickle cell trait removed.

Under the assumption of naturally acquired immunity, whereby individuals acquire protection against clinical malaria through repeated infections, we would expect age and/or the number of previously experienced malaria episodes to be correlated with a reduced risk of clinical malaria. Having access to individual episode histories we can thus analyse how both age and previous number of episodes alter the risk of future episodes. As illustrated in Figure 4, and against expectation, individuals who experience an episode in a given year are not different in age ( Figure 4A) but appear to have acquired more previous episodes on average than those who did not ( Figure 4B). Importantly, the fact that individuals with more episodes seem more at risk of a further episode does not seem to be an age effect but persists as individual grow older, which is demonstrated in Figure 5 by means of age-stratified distribution of previous number of episodes in individuals with and without a clinical episode.

### Birth time of year affects the risk of clinical malaria

**Figure 6.**
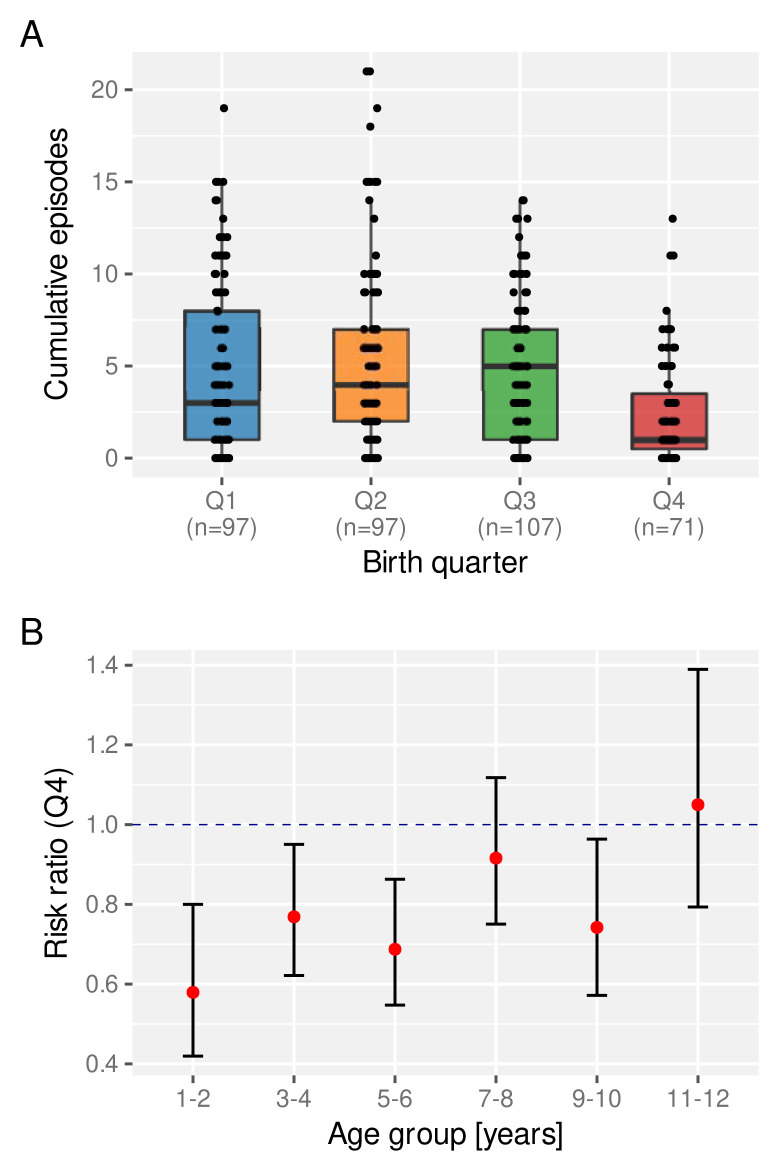
The effect of birth quarter on the risk of clinical malaria. **A**. Distribution of the number of clinical episodes experienced by an individual child by the age of 6 years stratified by birth quarter. Box and whisker plots indicate the median and inter quartile ranges. **B**. Risk ratio of experiencing a clinical episode in a given year between children born in Q4 compared to those born between Q1 and Q3.

Previous studies have shown that *in utero* exposure to *P. falciparum can affect a child’s future susceptibility to malaria (e.g.* 16– 19). Equally, poor nutrition during pregnancy has also been linked with long-term negative consequences for disease susceptibility (e.g. 20– 23. We therefore asked whether the birth time of year has an effect on a child’s experience of clinical malaria episodes. For this we looked at the total number of clinical episodes acquired by a certain age (here we set age = 6 years), stratified by birth quarter (Q1-Q4). As clearly indicated in Figure 6A, children born towards the end of the year (Q4) appear to be at a lower risk of a clinical episode on average than those who are born earlier in the year.

Next we investigated the temporal stability of this protective effect, for which we calculated the age-dependent risk ratio of children born in Q4 relative to those born in Q1–Q3. As can be seen in Figure 6B, Q4 children under the age of 6 years have a significantly reduced risk of experiencing a clinical malaria episode than those born earlier. This protective effect wanes as children get older, however, possibly under the influence of other environmental factors, including continuous exposure to the parasite.

### Risk factors of clinical malaria

The exploratory analyses presented above have revealed a high degree of heterogeneity in both malaria prevalence and in individual episode histories. In order to determine if and by how much the latter is simply a function of the former, and to what degree other factors have an independent effect on an individual’s risk of experiencing a clinical malaria episode we built a Bayesian hierarchical logistic regression model (see Methods). Specifically we investigated the effect of *PI*, *genotype* (AA/AS), age and birth quarter on the probability of a child experiencing a clinical episode in a given year.

**Figure 7.**
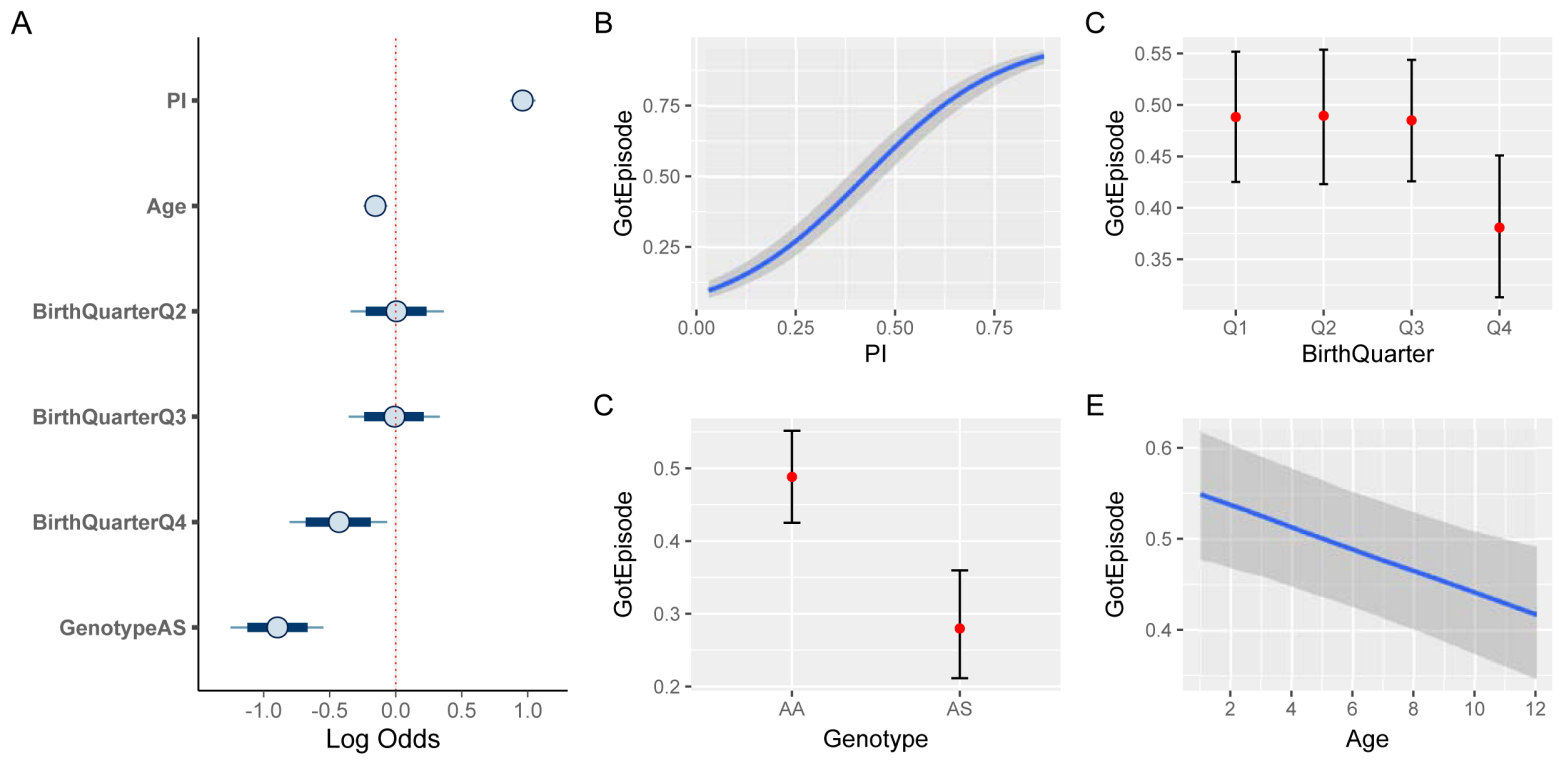
Risk factor analysis of clinical malaria. **A**. Estimated effect sizes (plus 80% and 95% CI; boxes and bars, respectively) for individual factors underlying risk of clinical malaria. **B**– **E**. Conditional effect plots showing the the probability of a clinical episode against prevalence index ( **B**), sickle cell genotype ( **C**), birth time of year ( **D**), and age ( **E**). The solid lines ( **B**, **E**) and circles ( **C**, **D**) represent the median and the shaded areas ( **B**, **E**) and whiskers ( **C**, **D**) the 95% prediction intervals.

Figure 7A shows the credible intervals (CI) of the regression coefficients (median plus 80% and 95% credible intervals). As expected, malaria prevalence ( *PI*) has the strongest effect on the risk of a clinical episode (median effect size estimate: 0.96, 95% CI [0.87, 1.06]). Equally expected is the protective effect of the sickle cell trait (-0.89, 95% CI [-1.23, -0.55]). In line with the above exploratory analysis ( Figure 6), birth quarter Q4 also has a protective effect (-0.42, 95% CI [-0.8, -0.03]). In contrast to the similarity in the age distribution between individuals who experience a clinical episode or not ( Figure 4A), accounting for inter-individual level variation we now find that older children have a slightly reduced risk of experiencing an episode compared to younger ones (-0.15, 95% CI [-0.24, -0.07]). Figure 7B–D illustrate the marginal effects of the four covariates on the probability of a clinical episode, again showing the protective effect of birth quarter Q4, the sickle cell trait (AS), and, to a smaller and more uncertain degree, age.

Our results suggest that beside exposure, by means of disease prevalence in the surrounding area ( *PI*), individual-level differences in susceptibility could have a significant effect on the risk of experiencing a clinical episode in a given year. Apart from the well-known protective sickle cell trait, these differences may stem from the birth time of year, for example, as demonstrated here, as well as other inter-individual differences accounted for by the hierarchical nature of our model (confirmed by means of model comparison against a non-hierarchical model based on the estimated log probability density, ELPD: -2136, hierarchical model, vs. -2326, non-hierarchical model; see Methods).

**Figure 8.**
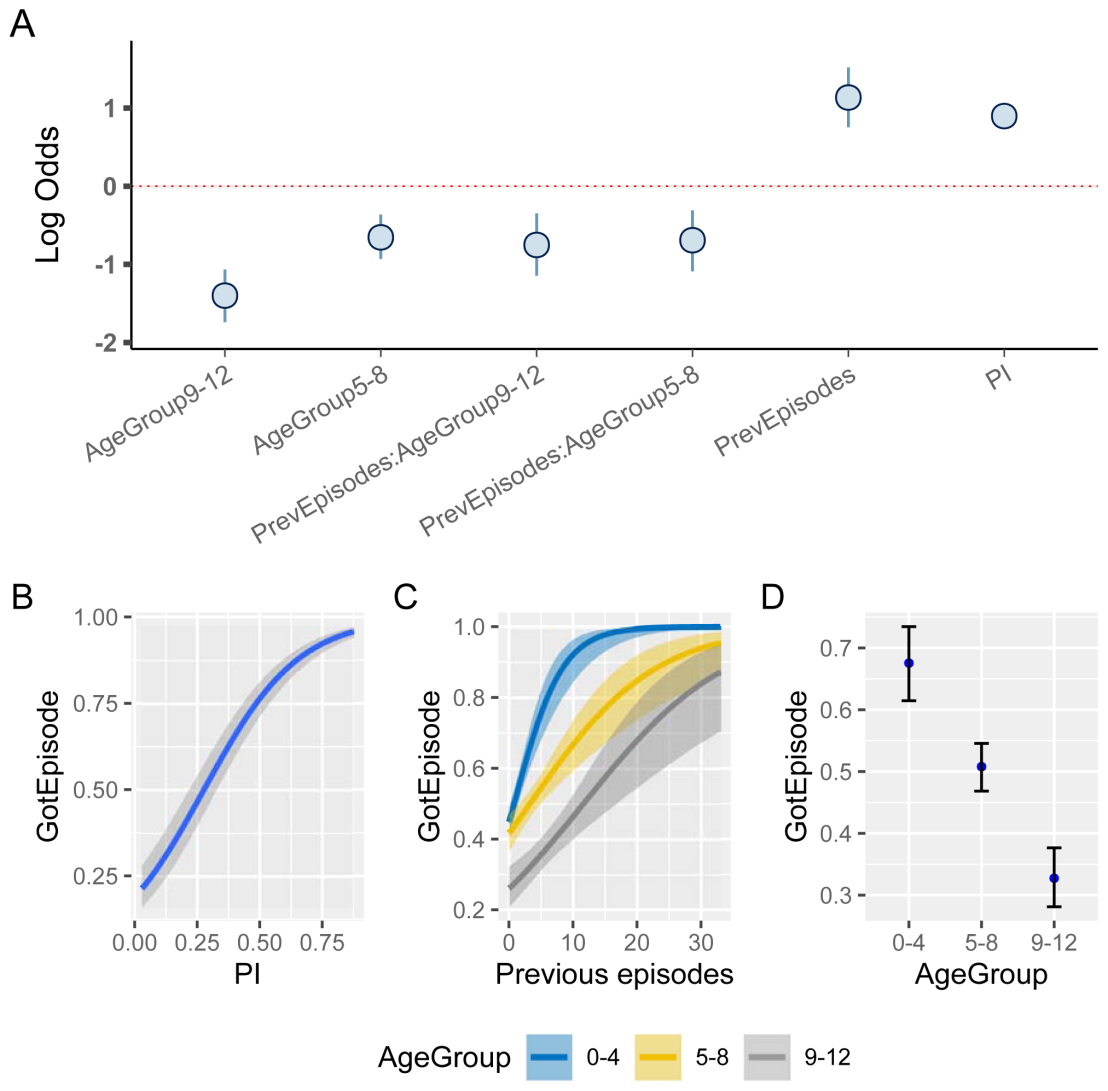
The effect of previous episode histories on the susceptibility to clinical malaria. **A**. Estimated effect sizes (plus 80% and 95% CI; boxes and bars, respectively) for individual factors underlying risk of clinical malaria. **B**– **D**. Conditional effect plots showing the the probability of a clinical episode against prevalence index ( **B**), previous episodes ( **C**), and age ( **D**). The solid lines and shaded regions represent the median and the 95% prediction intervals.

These individual-level differences, on the other hand, also imply that a child’s episode history could in fact be indicative of their risk of experiencing a future episode, which has already been alluded to in Figure 5. To quantify this further, we used a child’s previous number of clinical episodes as an explanatory variable in our model, alongside PI and Age. As the number of previous episodes is strongly influenced by the sickle cell trait, these children were removed from this analysis. As a reminder, all variables are centred (zero mean), which allows us to make inferences on the predicted changes in risk with respect to deviations from the variable’s mean value. Figure 8 demonstrates that children with an above average number of previous episodes are at a much higher risk of an additional episode than similar aged children with fewer episodes (median effect size estimate for 1–4 year old children: 1.14, 95%CI [0.79, 1.54]). Equally, older children appear to be more protected than younger children who have a similar number of previous episodes. In fact, accounting for the interaction between age and episode history we now also find that the effect of age is much more pronounced ( Figure 8C,D). The overall negative effect of the interaction between age and number of previous episodes therefore strongly suggests an independent protective effect of age, regardless of the previous number of episodes.

Taken together, our results are indicative of significant (inherent or induced) differences in the qualitative and quantitative nature by which children experience clinical malaria episodes and acquire clinical protection under repeated exposure to the parasite.

## Discussion

Here we used long-term epidemiological cohort data to investigate individual-level differences in children’s development of clinical protection against *P. falciparum* malaria. Our analyses reveal high levels of spatial and temporal heterogeneity in malaria incidence, which lead to significant differences in exposure levels to the parasite as children grow up in this cohort. However, we found that the variability in the rate at which individuals experience clinical episodes is not solely explained by exposure alone but points towards child-specific differences in disease susceptibility. We believe that these are important yet often neglected sources of variation in the data collected from prospective cohort studies aimed at identifying correlates of protection.

It has previously been reported that some children growing up in a malaria endemic region appear to suffer from excess malaria episodes compared to other children before reaching a similar state of clinical protection, even when accounting for exposure and age ^15^. These findings, which are broadly in line with the results presented here, add further evidence that children seem to exhibit inherent (phenotypic) differences in their susceptibility to clinical malaria episodes. Here we explicitly accounted for the inter-individual variability and concentrated primarily on an individual’s risk of experiencing a clinical episode in a given year or not. What was interesting to observe is that age itself only appeared to have a significant effect when accounting for this inter-individual variability. That is, although age usually needs to be factored in as a confounding factor when looking for immune correlates of protection, as older children are more likely to have experienced more episodes and have thus developed a higher degree of protection, we find that in this relatively low transmission setting and below the age of 12 years it only has a marginal effect on someone’s risk of experiencing a clinical episode.

Unsurprisingly, the most significant risk factor was simply exposure. Here we devised a new prevalence index, which provided a measure of infection prevalence in the surrounding area in a given year. It is related to the exposure index as previously introduced by Olotu *et al.* ^9^ but yields more spatially smoothed estimates and in this case revealed clear temporal trends in the spatial distribution of infection prevalence ( Figure 2). Although our calculation of the prevalence index was based only on children with a confirmed episode, it is unlikely that these heterogeneities disappear when accounting for all individuals. Another important point to make here is that this measure is semi-quantitative in that it is based on the number of infected children, and not the total number of infections in the surrounding area. However, and as evidenced by our analyses, it is strongly correlated with the probability of a child experiencing a clinical episode in a given year and thus captures the level of malaria transmission in the surrounding area. Our findings are also in line with previous studies that have revealed that malaria infections are not necessarily homogeneous across space but can exhibit pronounced hotspots of variable stability (e.g. 12, 13, 24). Unfortunately, the data analysed here did not allow us to investigate whether these spatio-temporal patterns are also reflective of locally changing transmission intensities or are purely driven by the changing immunity landscape among the study participants, or possibly both.

Apart from age and exposure, our analysis suggests that the time of year when an individual is born has a protective effect against clinical episode and that this effect can last for many years of early childhood. In this case we found that individuals born during the last quarter of the year (October - December) had a reduced risk of experiencing clinical episodes, which also manifested in a lower number of total episodes compared to other age-matched children under similar exposure conditions. In terms of timing, being born during Q4 in this cohort means that infants may acquire their first infection at the age of 4–6 months towards the end of maternal protection, assuming that the main transmission season in Junju starts around April. Alternatively, it might open up the possibility that their mothers got infected during the second trimester. It has previously been reported that malaria infections during pregnancy can alter an infant’s susceptibility to clinical malaria (e.g. 16– 19). It is also known that nutritional intake during pregnancy, which in many parts of the world still changes significantly between seasons, can have a lasting effect on the child’s susceptibility to disease (e.g. 21). Unfortunately, we cannot say whether the effect that we observe is due to *in utero* exposure during pregnancy or first exposure under waning maternal protection. Follow-up studies will be required to answer both this question and also if similar effects can be found in other malaria endemic settings.

Another important finding is that the risk of a clinical malaria episode is not simply a decreasing function of the number of episodes experienced in the past. In fact, we found that those children who have experienced many more episodes by a certain age have a much higher baseline risk than other, similar aged children. What we cannot conclude from this analysis is whether this might be due to inherent, i.e. genetic differences in susceptibility to clinical malaria, or whether these differences are somehow induced whereby an exposure to the parasite early in life can lead to impaired immunity to malaria, which in turn leads to further episodes of malaria in the future. A recent study has also found that individuals who carry asymptomatic infections are at an increased risk of experiencing a symptomatic episode in the near future ^25^. However, as the observed effect declined rapidly over time and was also seen across all age groups it is questionable whether it describes the same phenomenon as reported here. Using a systems immunological approach, we have previously described how repeated malaria episodes can lead to measurable modifications of the immune system, based on the comparison between children with few versus a large number of episodes ^26^. Using the insights gained from this analysis it would thus be interesting to follow a similar approach and compare children based on the risk factors identified herein. 

There are a number of caveats in this study. The first is that we only monitored children under the age of 12 years. What is clear from our analyses is that by this age, and in this setting, many of the children are still experiencing clinical episodes at a rate similar to younger children, even though their risk of severe and life-threatening malaria had declined long before that. Due to limited sample sizes and high variability in malaria transmission over space and time, identifying children who have acquired a level of natural protection is almost impossible simply by comparing incidence of clinical malaria. In fact, even children who appeared to have *plateaued* in experiencing new episodes ^24^ and are thus believed to have acquired a state of clinical protection can succumb to a malaria episode once more in years of high transmission (e.g. 2018). This, on the other hand, is an important point to re-emphasize: protection itself is not a dichotomous state but can best be understood as the probability of developing clinical symptoms if infected. Monitoring individual children and their level of exposure to malaria over long periods of time is therefore required to robustly infer immunological changes underlying their transition to clinical protection. The other caveat is that our measure of prevalence is retrospective, i.e. it is a summary measure based on all recorded infections over the entire year. One could thus argue that its high correlation with clinical episodes follows a circular argument. However, as the index case was not included in its calculation and because its computation is done over the same period of time as the response (clinical episode in that year, yes or no), this should not be a reason for concern.

## Conclusions

Cohort studies and results based on them are often affected by the high variability in the underlying data. This may not only drown out real effects, which can be small even when comparing clearly define phenotypes at the gene expression level ^25^, but can also make comparisons between studies challenging. What we have demonstrated here is that individual-level variations in observed outcomes, in this case the incidence of clinical episodes in a given year, are more than noise in the collected data and have to be taken into consideration when trying to make inferences from population-level measures. The highlighted heterogeneity in exposure to the parasite implies that children experience different transmission intensities at different times during their life. So far we do not know whether there is a (long-term) difference in children who experience the majority of their clinical episodes before the age of 6 years or after, for example. What is clear, though, is that there are pronounced differences in the children’s pathway to becoming clinically protected. Identifying those who get there much faster than others might be key for broadening our understanding of natural acquired protection against malaria.

## Data availability

### Underlying data

Kemri Wellcome Trust Research Programme (KWTRP) Research Data Repository: Replication Data for: Individual-level variations in malaria susceptibility and acquisition of clinical protection, ( https://doi.org/10.7910/DVN/WQCKJJ) ^27^.

Note, spatial location data of individual homesteads has been removed due to confidentiality reasons. Data sharing requests will be reviewed by the Data Governance Committee at KEMRI CGMRC Kilifi ( dgc@kemri-wellcome.org) who will manage the process and ensure that appropriate ethical approval is in place (if applicable), consent obtained caters for uses outlined in the data request, and the request is in line with the institution’s policies on data sharing.

### Extended data

Kemri Wellcome Trust Research Programme (KWTRP) Research Data Repository: Replication Data for: Individual-level variations in malaria susceptibility and acquisition of clinical protection, ( https://doi.org/10.7910/DVN/WQCKJJ) ^27^.

This project contains the following extended data:

- Data overview
- Statistical analysis

Data are available under the terms of the Creative Commons Attribution 4.0 International license (CC-BY 4.0).
