# Supplementary material for: Individual-level variations in malaria susceptibility and acquisition of clinical protection
Source: Wellcome Open Res. 2022 Feb 25;6:22. Originally published 2021 Feb 2. [Version 3] doi: 10.12688/wellcomeopenres.16524.3 (PMC8914138; doi:10.12688/wellcomeopenres.16524.3)
Supplement: Peer review history

# Peer review: Reviewer response for version 3

**DOI:** 10.21956/wellcomeopenres.19562.r48907

**Published:** 2022-03-10

**Authors:** Wendy Prudhomme-O'Meara, Christine Markwalter

**Affiliations:** 1 Duke Global Health Institute, Duke University, Durham, NC, USA; 2 Moi University College of Health Sciences, Eldoret, Kenya

The authors considered our comments and made useful additions to the methods in particular. It is disappointing that most of their responses to our more technical comments will remain hidden in the reviewer dialogue. Some of these limitations seem to be related to the very lengthy review processes and restrictions of the journal. I still feel that fitting the parameter 'a' to maximize the correlation of PI is a bit circular. They are basically fitting PI to the outcome and then finding that it’s a risk factor. 

 Also, please consider the following to check for a typo: 

-  Pg 4, when discussing the spatial weighting factor a: “The spatial weighting factor *a* determines how rapidly the influence of an infection at *j* on the prevalence at *j* declines with increased spatial distance.” I’m pretty sure they mean: “…influence of an infection *j* on the ***PI*** at ***i*** …”

Is the work clearly and accurately presented and does it cite the current literature?

Partly

If applicable, is the statistical analysis and its interpretation appropriate?

Partly

Are all the source data underlying the results available to ensure full reproducibility?

Partly

Is the study design appropriate and is the work technically sound?

Partly

Are the conclusions drawn adequately supported by the results?

Yes

Are sufficient details of methods and analysis provided to allow replication by others?

No

Reviewer Expertise:

Malaria, epidemiology, implementation science/trials

We confirm that we have read this submission and believe that we have an appropriate level of expertise to confirm that it is of an acceptable scientific standard.

# Peer review: Reviewer response for version 2

**DOI:** 10.21956/wellcomeopenres.19005.r47493

**Published:** 2022-01-26

**Authors:** Wendy Prudhomme-O'Meara, Christine Markwalter

**Affiliations:** 1 Duke Global Health Institute, Duke University, Durham, NC, USA; 2 Moi University College of Health Sciences, Eldoret, Kenya

This manuscript presents an analysis of how individual differences in exposure affect children’s risk of experiencing a clinical malaria episode. Based on weekly surveillance in a 12-year longitudinal birth cohort in Kenya, the authors describe spatio-temporal variability in malaria incidence and heterogeneity in clinical episode histories for children living in a relatively small geographic area. They found that children with a high number of previous clinical episodes are at a higher risk of an additional episode, challenging the expectation that protective immunity, and therefore decreased risk of clinical episode, is acquired with increasing symptomatic exposure. While the manuscript is well-written and of interest to the malaria community, we have some concerns regarding the metrics used to define local exposure and potential sampling bias, which are detailed below.

 Major concerns:

 The Prevalence Index (PI) was described in the manuscript as capturing the spatial distribution of malaria over time within the cohort. It was also found to have the strongest effect on risk of clinical malaria compared to other covariates. However, we are concerned:

- PI is used as a metric of local risk, but it is not clear how spatially representative the cohort is. There is no information about intervening households not enrolled in the cohort. PI assigns a number to only the local malaria cases only among cohort members which may not reflect something fundamental about the underlying epidemiology. It would be helpful to comment on how spatially representative cohort households are in Junju or how children were selected for enrollment. We went back to some previous papers and still could not quite get this clearly.
- Some clarification of the variable definitions for PI is needed. The introduction to PI in the methods describes summing over all recorded malaria episodes in a year; however, the sums are defined over *j*, which is described as a location in the text. If the latter, and since *Z _j,t_* is binary for at least one clinical episode at location *j*, locations/households with vastly different rates of clinical malaria in a given year would be weighted equally, diminishing some heterogeneity in the data.
- Given the previous two points, and particularly since this number is based on symptomatic cases, we feel that the term “prevalence” index could be misleading. “Cohort incidence index” or similar may be more appropriate.
- It would be useful to see more information about ‘a’. It is given as 2 with an indication that it was estimated from the data, but more information about *a*, a description of how well *a* fit the data, a sensitivity analysis of the effect of *a* on the relationship between PI & episodes all would be useful in understanding this central parameter.
- It is interesting that the zones of highest ‘PI’ seem to shift from north to central to south over the course of the study period. This is consistent with our finding of dynamic hotspots of fever (Platt Sci Rep). Could this be related to local levels of population immunity? Or is it influenced by the open cohort model where you have more cohort members in different zones over time?

Regarding the birth exposure analysis, is it possible that Q4 births are at lower risk simply because they have less accumulated exposure/episodes before entering the cohort? Given the incremental differences between Q1--> Q2 -->Q3-->Q4, with the largest difference seen between Q1 and Q4 births, and the effect waning with age, this seems like one possible explanation. Based on your annualized outcome (Gotepisode = annual have episode or not), I assume that a child enters the analysis only for the first calendar year AFTER their first birthday which could introduce the above bias. With the detailed data available, could a more precise metric of pregnancy exposure be estimated, based on rainfall, total cohort cases in the 9 months before their birth, or even PI associated to a birth (or pregnancy). This would be a more compelling comparison.

 Other concerns:

- Did you observe any household clustering of children with high numbers of cumulative episodes? Our work has demonstrated that infections are primarily clustered (Obala PLOS One) and also related (Nelson Nat Comm) at the household level (as measured by haplotype sharing) indicating that spatial structure of local ‘prevalence’ beyond the household is probably measuring a risky environment (ie mosquito density) rather than the actual risk of a nearby infection on another susceptible individual. Additional consideration/discussion of household-level factors would strengthen the overall conclusions of the manuscript.
- Figure 5: The y axis values (0 - 3.5 previous episodes) in Figure 5 do not seem to agree with those in Figures 3 and 4B, where it appears some children experienced up to 30 previous episodes. How do you reconcile these numbers?
- Given the large differences between years in overall episodes, shouldn’t year be a covariate in the model given that children will have episode data spanning years with much different overall morbidity? In a previous analysis that included this cohort, calendar year was identified as a significant independent predictor of clinical malaria (Ref 15; Ndungu *et al*. BMC Med 2015).
- Why was *PrevEpisodes* excluded from the risk factor analysis in Figure 7?
- Figure 8: It would be helpful to have a graphical representation of the interaction between previous episodes and age to aid the reader in understanding your interpretation of the interaction as “older individuals will by nature have experienced more episodes and also acquired a higher degree of protection”; for example, plotting Fig 8C for multiple ages/age strata would more clearly communicate your point.
- Reading your responses to Dr. Okell’s thoughtful comments significantly aided in our understanding of different aspects of the manuscript; however, some of the indicated modifications still seem to be missing from version 2 of the manuscript, including:
  - Revised description of spatial homo/heterogeneity in results section text for Fig 2.
  - Additional panel in Fig 4 showing the distribution of incidence (clinical episodes per year) for all children against age.
  - Clarifying the units to effect sizes/regression coefficients for Fig 7-8
  - Additional interpretation in the text for Figure 8C and 8D
- Other studies have also found that previous exposure increases risk of future exposure (Sumner eLIFE, asympt exposure increases risk of future symptoms).

Is the work clearly and accurately presented and does it cite the current literature?

Partly

If applicable, is the statistical analysis and its interpretation appropriate?

Partly

Are all the source data underlying the results available to ensure full reproducibility?

Partly

Is the study design appropriate and is the work technically sound?

Partly

Are the conclusions drawn adequately supported by the results?

Yes

Are sufficient details of methods and analysis provided to allow replication by others?

No

Reviewer Expertise:

Malaria, epidemiology, implementation science/trials

We confirm that we have read this submission and believe that we have an appropriate level of expertise to confirm that it is of an acceptable scientific standard, however we have significant reservations, as outlined above.

# Response

**Published:** 2022-01-31

**Authors:** Mario Recker

**Affiliations:** Department of Mathematics, University of Exeter, UK

We would like to thank the reviewers for their careful evaluation of our manuscript, which we hope has helped to improve clarity regarding the methodology, incl. sampling, and presentation of our results. Please see below for a point-by-point response to all concerns raised by the reviewers. Note, we have included here some additional figures to address specific points; these are not part of the manuscript as we felt they would distract from the main findings.

**Major concerns:**

 The Prevalence Index (PI) was described in the manuscript as capturing the spatial distribution of malaria over time within the cohort. It was also found to have the strongest effect on risk of clinical malaria compared to other covariates. However, we are concerned:

- PI is used as a metric of local risk, but it is not clear how spatially representative the cohort is. There is no information about intervening households not enrolled in the cohort. PI assigns a number to only the local malaria cases only among cohort members which may not reflect something fundamental about the underlying epidemiology. It would be helpful to comment on how spatially representative cohort households are in Junju or how children were selected for enrollment. We went back to some previous papers and still could not quite get this clearly.

***Response:** This is an important point. Recruitment into the cohort was based on which homesteads volunteered following sensitisation of the whole area and was not subject to specific spatial or demographic selection criteria. We therefore believe that the cohort analysed here does provide an unbiased spatial representation of the village as a whole and have added this information in our revised manuscript (Methods section). Furthermore, although the prevalence index (PI) is based on recorded malaria episodes in only a small subset of the population, it strongly correlates with an individual’s risk of malaria in a given year. And whilst we agree that this simple index does not capture all of the underlying factors contributing to risk, we cannot think of any reasons why it would not be reflective of the general spatio-temporal epidemiology in this area.*

  

- Some clarification of the variable definitions for PI is needed. The introduction to PI in the methods describes summing over all recorded malaria episodes in a year; however, the sums are defined over *j*, which is described as a location in the text. If the latter, and since *Z _j,t_* is binary for at least one clinical episode at location *j*, locations/households with vastly different rates of clinical malaria in a given year would be weighted equally, diminishing some heterogeneity in the data.

***Response:** We agree that the description of how the prevalence index is derived lacked clarity, which we hope is now easier to follow in the revised version. In brief, for each individual we sum over all individuals (not homesteads) within a certain spatial neighbourhood (radius around the index location). For this we used a binary classification: 1 if the neighbouring individual had a recorded episode that year, and 0 otherwise. This way, locations or households with vastly different rates of clinical malaria, that the reviewers were alluding to, will be weighted differently; that is, a location / household with many individuals who had a recorded infection contribute more than an equally distant location with only a single individual. Spatial heterogeneity can therefore be captured whilst also achieving some spatial smoothing.*

  

- Given the previous two points, and particularly since this number is based on symptomatic cases, we feel that the term “prevalence” index could be misleading. “Cohort incidence index” or similar may be more appropriate.

***Response: **This is a fair point; however, at this stage of the reviewing process, with the manuscript having been online for over a year, we feel it might be better to stick with prevalence index but to clarify this point in the revised manuscript to avoid potential misinterpretation.*

  

- It would be useful to see more information about ‘a’. It is given as 2 with an indication that it was estimated from the data, but more information about *a*, a description of how well *a* fit the data, a sensitivity analysis of the effect of *a* on the relationship between PI & episodes all would be useful in understanding this central parameter.

***Response:** This is another important point, which also lacked detail in the original submission. The parameter a acts like spatial smoothing, with larger values corresponding to smaller influences of spatially distant locations contributing to the prevalence index (PI), leading to more spatial heterogeneity, as shown in the graph below plotting the standard deviation of PI against a.*

Graph 1 

*We initially tried to use spatial statistics to infer the value from the data but found that it varied too much between individual years. In the end we therefore decided to use a value that maximised the correlation between PI and number of episodes for all individuals and all years, as shown in the graph below.*

Graph 2

*This value was also found to be a good ‘visual’ compromise between spatial smoothing and maintaining sufficient heterogeneity to demonstrate spatio-temporal trends (Fig. 2). We would usually add these graphs as supplementary material but here, given the WT OpenResearch publishing format, felt that they would distract from the main findings. We hope that interested readers will still be able to find this information in the comments section.*

  

- It is interesting that the zones of highest ‘PI’ seem to shift from north to central to south over the course of the study period. This is consistent with our finding of dynamic hotspots of fever (Platt Sci Rep). Could this be related to local levels of population immunity? Or is it influenced by the open cohort model where you have more cohort members in different zones over time?

***Response:** We agree that this was an interesting finding and partly motivated some of the subsequent analyses. We speculate that this might indeed be driven by a dynamically changing immunity landscape, as previously indicated in Bejon et al. PloS Med (2010) and also in line with the paper referred to by the reviewers, rather than spatially biased enrolment into the cohort (for reasons mentioned above). We have added a brief discussion on this point in our revised manuscript.*

 Regarding the birth exposure analysis, is it possible that Q4 births are at lower risk simply because they have less accumulated exposure/episodes before entering the cohort? Given the incremental differences between Q1--> Q2 -->Q3-->Q4, with the largest difference seen between Q1 and Q4 births, and the effect waning with age, this seems like one possible explanation. Based on your annualized outcome (Gotepisode = annual have episode or not), I assume that a child enters the analysis only for the first calendar year AFTER their first birthday which could introduce the above bias. With the detailed data available, could a more precise metric of pregnancy exposure be estimated, based on rainfall, total cohort cases in the 9 months before their birth, or even PI associated to a birth (or pregnancy). This would be a more compelling comparison.

***Response:** This is an interesting hypothesis but there are reasons to believe why this cannot fully explain our observations. Children are enrolled at birth but for our analyses we only selected children 1 year old or above. It is true that individuals born early January have nearly an extra year of exposure compared to those born late in December and therefore could theoretically have more cumulative episodes. However, we did not find an incremental differences between Q1->Q2->Q3->Q4 that the reviewers are referring to (see Figs. 6 and 7). This becomes more apparent when we stratified by birth month, as shown below (due to low sample sizes we a birth quarter stratification in the manuscript).*

Graph 3

*Furthermore, although it is possible that the discrete nature of age and year can introduce the referred bias in the number of cumulative episodes, this would not affect their risk of getting a clinical episode in subsequent years, which our analysis showed was reduced for those born in Q4. We fully agree that these observations warrant more detailed analyses into the likely causes (e.g. exposure during pregnancy or exposure during first year of life), and in fact is something that we are currently working on, but we feel that this was outside the scope of this manuscript.*

**Other concerns: **

- Did you observe any household clustering of children with high numbers of cumulative episodes? Our work has demonstrated that infections are primarily clustered (Obala PLOS One) and also related (Nelson Nat Comm) at the household level (as measured by haplotype sharing) indicating that spatial structure of local ‘prevalence’ beyond the household is probably measuring a risky environment (ie mosquito density) rather than the actual risk of a nearby infection on another susceptible individual. Additional consideration/discussion of household-level factors would strengthen the overall conclusions of the manuscript.

***Response:** We did not look specifically at household clustering but agree that this might be a potentially important factor contributing to an individual’s risk of infection / clinical episodes. Of a total of over 500 individuals analysed, only around 50 shared homesteads, and because we only considered children aged 1-12 years, rather than all members in a household, we do not believe that this would make a significant difference to the results presented here.*

  

- Figure 5: The y axis values (0 - 3.5 previous episodes) in Figure 5 do not seem to agree with those in Figures 3 and 4B, where it appears some children experienced up to 30 previous episodes. How do you reconcile these numbers?

***Response:** Thank you for pointing this out, this was due to an unfortunate mislabelling of the y-axes; what was plotted in Fig. 5 was the average number of episodes per year, i.e. cumulative episodes / age. We have now revised this figure to show total number of previous infections (going up to >30 in the 12 year old age class).*

  

- Given the large differences between years in overall episodes, shouldn’t year be a covariate in the model given that children will have episode data spanning years with much different overall morbidity? In a previous analysis that included this cohort, calendar year was identified as a significant independent predictor of clinical malaria (Ref 15; Ndungu *et al*. BMC Med 2015).

***Response:** This is a good point; however, the effect of year is implicitly taken care of by the prevalence index, which strongly correlates with the total number of episodes and hence year, as shown in the below graph.*

Graph 4

  

- Why was *PrevEpisodes* excluded from the risk factor analysis in Figure 7?

***Response:** The reason why PrevEpisodes was excluded is because we argued that individual-level factors such as genotype or birth quarter will affect the number of previous episodes experienced by a child, i.e. PrevEpisodes itself will be a function of those factors. This is why we then carried out the subsequent analysis using only PrevEpisodes and PI as covariates, which is shown in Fig. 8.*

  

- Figure 8: It would be helpful to have a graphical representation of the interaction between previous episodes and age to aid the reader in understanding your interpretation of the interaction as “older individuals will by nature have experienced more episodes and also acquired a higher degree of protection”; for example, plotting Fig 8C for multiple ages/age strata would more clearly communicate your point.

***Response:** We fully agree and have now revised the analysis to use age categories instead of using age as a continuous variable. The new figure also more clearly demonstrates the interaction effect between the number of previous episodes and age (i.e. that the effect size of PrevEpisodes decreases with increasing age).*

Graph 5

  

- Reading your responses to Dr. Okell’s thoughtful comments significantly aided in our understanding of different aspects of the manuscript; however, some of the indicated modifications still seem to be missing from version 2 of the manuscript, including:
  - Revised description of spatial homo/heterogeneity in results section text for Fig 2.
  - Additional panel in Fig 4 showing the distribution of incidence (clinical episodes per year) for all children against age.
  - Clarifying the units to effect sizes/regression coefficients for Fig 7-8
  - Additional interpretation in the text for Figure 8C and 8D

***Response:** We apologise for this but are equally puzzled as to why none of our resubmitted and revised material (manuscript and figures) following Dr. Okell’s comments had been available online. We will try to navigate this system more carefully to make sure that all updated text and figures are available for review.*

  

- Other studies have also found that previous exposure increases risk of future exposure (Sumner eLIFE, asympt exposure increases risk of future symptoms).

***Response:** Thank you for pointing us towards this interesting study, which incidentally was published after our manuscript went online. We would like to point out that the effect described therein declined rapidly over time and was observed across all age groups. It is therefore questionable whether this describes the same phenomenon underlying our observations. This is now briefly discussed in the Discussion.*

# Peer review: Reviewer response for version 2

**DOI:** 10.21956/wellcomeopenres.19005.r45823

**Published:** 2021-09-14

**Authors:** Lucy C. Okell

**Affiliations:** 1 MRC Centre for Global Infectious Disease Analysis, Department of Infectious Disease Epidemiology, Imperial College London, London, UK

The authors have addressed all my comments very thoroughly - thank you for the insightful responses. I have no further comments.

Is the work clearly and accurately presented and does it cite the current literature?

Yes

If applicable, is the statistical analysis and its interpretation appropriate?

Partly

Are all the source data underlying the results available to ensure full reproducibility?

Yes

Is the study design appropriate and is the work technically sound?

Yes

Are the conclusions drawn adequately supported by the results?

Yes

Are sufficient details of methods and analysis provided to allow replication by others?

Yes

Reviewer Expertise:

Epidemiology, mathematical modelling

I confirm that I have read this submission and believe that I have an appropriate level of expertise to confirm that it is of an acceptable scientific standard.

# Peer review: Reviewer response for version 1

**DOI:** 10.21956/wellcomeopenres.18203.r44305

**Published:** 2021-07-13

**Authors:** Lucy C. Okell

**Affiliations:** 1 MRC Centre for Global Infectious Disease Analysis, Department of Infectious Disease Epidemiology, Imperial College London, London, UK

This analysis harnesses a rich dataset following children over many years within one area and assessing the incidence of clinical malaria episodes. The findings are very interesting and challenge the idea that immunity to disease could be acquired after a specific number of previous disease episodes. Disease appears to be more concentrated within particular individuals than might be expected within a given area. The findings have important implications for studies of malaria susceptibility, risk factors, immune protection, and mathematical models. The analysis is detailed, thoughtful and well written.

 I have some comments and clarifications:

- Could you clarify in the methods whether children were continuously recruited during the study period, or were all recruited at birth at the beginning of the study and followed up? This becomes clearer later on, but it would help interpretation e.g. of Figure 1, where it’s not quite clear if year also indicates increasing average age of the cohort.
- Can you briefly state whether any interventions were provided as part of the cohort study (treatment, nets…)
- Are all episodes uncomplicated malaria, or do you count severe malaria also?
- Statistical modelling – can you explain a bit more the ‘got episode’ outcome versus the ‘previous episodes’ explanatory variable. Does ‘got episode’ depend on the most recent year of data for a given child, while the previous episodes are those occurring in the years before that?
- In this part of the results: “in those years we not only see more individuals with a clinical episode but a disproportional increase in the numbers of episodes per individual” – how is the disproportional part assessed – maybe based on a Poisson/negative binomial distribution or similar?
- Figure 2 is a nice depiction. Are the years with high prevalence definitely more spatially homogeneous? Visually the lower prevalence years, e.g. 2007 and 2012 also look quite homogeneous.
- Fig 4 is very interesting. Why are sickle cells carriers removed from panel B but not A?
- It could be helpful to have a standard plot of incidence of episodes by age near the beginning, to help the reader compare this cohort to those from other endemic settings.
- P6 Can you clarify the units of the effect size estimates - are they the regression coefficients on the log odds scale? i.e. 0 = no effect? Same in the fig 7A legend.
- Fig 7 – I like these plots, but is the blue just 1-yellow? It may be clearer with just one or the other. It would be good to also define ‘GotEpisode’ again in the legend to save the reader referring back – i.e. probability of a current clinical episode – per year, I think?
- Fig 8. Given the interaction, more explanation is needed for panels A, C and D. What is the baseline group for age and previous episodes in the regression, and what units are used? For what age does C show the effect of previous episodes? (the youngest age perhaps, or the median?). It could be helpful to plot panel C twice, once the effect of previous episodes for a younger age and once for an older age, so the reader has more intuition how the interaction works (there should be less effect of previous episodes at older ages given the direction of interaction?). Does the effect of previous episodes ever become protective at older ages in the multivariable model? (despite not being protective in Fig 5).
- Same query for panel D – for what number of previous episodes does this age effect apply?
- Can you comment on the strength of correlation between previous episodes and the prevalence index? Is it possible to distinguish the two effects from each other during the regression? E.g. can a child with say 20 previous episodes come from a lower PI area?
- A very interesting discussion. Could you explain this sentence in the last paragraph some more? “children experience different transmission intensities at different times during their life” – I was not sure how this was inferred.

Is the work clearly and accurately presented and does it cite the current literature?

Yes

If applicable, is the statistical analysis and its interpretation appropriate?

Partly

Are all the source data underlying the results available to ensure full reproducibility?

Yes

Is the study design appropriate and is the work technically sound?

Yes

Are the conclusions drawn adequately supported by the results?

Yes

Are sufficient details of methods and analysis provided to allow replication by others?

Yes

Reviewer Expertise:

Epidemiology, mathematical modelling

I confirm that I have read this submission and believe that I have an appropriate level of expertise to confirm that it is of an acceptable scientific standard, however I have significant reservations, as outlined above.

# Response

**Published:** 2021-09-03

**Authors:** Mario Recker

**Affiliations:** Department of Mathematics, University of Exeter, UK

We would like thank the reviewer for their positive evaluation of our work and critical comments, which we believe has helped to improve the quality and readability of our manuscript. Please see below for a point-by-point response to issues raised.

  

- *Could you clarify in the methods whether children were continuously recruited during the study period, or were all recruited at birth at the beginning of the study and followed up? This becomes clearer later on, but it would help interpretation e.g. of Figure 1, where it’s not quite clear if year also indicates increasing average age of the cohort.*
**Response:** Since the initial setup of this cohort in 2005, children have been continuously recruited at or shortly after birth and actively monitored on a weekly basis for detection of malaria episodes. Furthermore, we aimed to maintain a similar average age over time by only selecting children that were between 1 and 12 years old in any given year. We have clarified this in the Method section.
- *Can you briefly state whether any interventions were provided as part of the cohort study (treatment, nets…)*
**Response:** This was unfortunately omitted in the description of the data / cohort. All malaria positive children are treated with co-artemether. Bednet coverage is high in this cohort (doi: 10.1186/s12879-017-2822-x) and hence we did not consider it necessary to distribute nets specifically to study participants.  We found no correlation between bednet use and outcome; hence bednet use was not considered for further analyses. This has now been added to the Methods section.
- *Are all episodes uncomplicated malaria, or do you count severe malaria also? *
**Response:** This community based active surveillance of malaria with prompt provision of anti-malarials did not identify episodes of severe malaria. A malaria episode (which relates to our response variable *GotEpisode*) is defined as axillary temperature ≥37.5°C associated with >2,500 *P. falciparum* parasites per microliter of blood. This has been clarified.
- *Statistical modelling – can you explain a bit more the ‘got episode’ outcome versus the ‘previous episodes’ explanatory variable. Does ‘got episode’ depend on the most recent year of data for a given child, while the previous episodes are those occurring in the years before that?*
**Response:** As outlined in the Methods section, *GotEpisode* is a binary response variable (yes/no) indicating whether an individual experienced a clinical episode (defined as above) in a given year or not. The variable *PrevEpisodes*, on the other hand, is the recorded total number of episodes a child has experienced up until but not including the year in question. We have tried to make this clearer in the Method section. 
- *In this part of the results: “in those years we not only see more individuals with a clinical episode but a disproportional increase in the numbers of episodes per individual” – how is the disproportional part assessed – maybe based on a Poisson/negative binomial distribution or similar? *
**Response:** This was originally assessed by eye only as it was clear from Figure 1B that in years with low-to-medium levels of infections, less than a third of children experience more than 2 episodes per year, whereas this increases to around two thirds in years of high prevalence. We have now added another panel showing the frequency distribution of 1, 2, and 3+ episodes to the graph to make this point clearer. 
- *Figure 2 is a nice depiction. Are the years with high prevalence definitely more spatially homogeneous? Visually the lower prevalence years, e.g. 2007 and 2012 also look quite homogeneous.*
**Response:** This is a good point and unfortunate wording on our part. We have replaced this sentence with “ *Furthermore, we find that in high prevalence years (2014 and 2018), malaria incidence was not confined to particular areas but was observed almost across the entire cohort, which could be indicative of more intense and/or longer transmission seasons.*”
- *Fig 4 is very interesting. Why are sickle cells carriers removed from panel B but not A?*
**Response:** The reason for removing sickle cell carriers in Figure 4B is because it is a known protective trait. AS children will therefore have a lower total number of previous episodes and are also less likely to experience an episode in a given year, such that leaving them in would confound the results. On the other hand, they would not influence the relationship between age and the risk of an episode. We have added a note on this in the main text.    
- *It could be helpful to have a standard plot of incidence of episodes by age near the beginning, to help the reader compare this cohort to those from other endemic settings. *
**Response:** Thank you for the suggestion. We have now added an additional panel to Fig 4 showing the distribution of incidence (clinical episodes per year) for all children against age. This graph demonstrates that age on its own, at least in this cohort within the age range considered, is not a good predictor of clinical protection.  
- *P6 Can you clarify the units of the effect size estimates - are they the regression coefficients on the log odds scale? i.e. 0 = no effect? Same in the fig 7A legend. *
**Response:** All continuous variables were centred and scaled for the statistical analyses to permit better comparison between effect sizes. For those variables a unit represents one standard deviations from the mean. And yes, the regression coefficient are log odds, with 0 indicating no effect, negative values a protective effect and positive values an increase in risk. We have added a corresponding note to the Methods section and the main text and changed the x-axis labels in Figs 7A and 8A.  
- *Fig 7 – I like these plots, but is the blue just 1-yellow? It may be clearer with just one or the other. It would be good to also define ‘GotEpisode’ again in the legend to save the reader referring back – i.e. probability of a current clinical episode – per year, I think? *
**Response:** Thank you for the suggestion, we have amended the figure accordingly (as blue was indeed 1-yellow). We have also added a reminder to the main text and figure legends that *GotEpisode* refers to a child experiencing a clinical malaria episode in a given year or not. 
- *Fig 8. Given the interaction, more explanation is needed for panels A, C and D. What is the baseline group for age and previous episodes in the regression, and what units are used? For what age does C show the effect of previous episodes? (the youngest age perhaps, or the median?). It could be helpful to plot panel C twice, once the effect of previous episodes for a younger age and once for an older age, so the reader has more intuition how the interaction works (there should be less effect of previous episodes at older ages given the direction of interaction?). Does the effect of previous episodes ever become protective at older ages in the multivariable model? (despite not being protective in Fig 5).*
**Response:** We fully agree that Fig 8 was hard to interpret without further explanation. As mentioned above, all numerical variables are centred and scaled, so the effects shown in panel A are with respect to a standard deviation from the mean. Panel B is probably the easiest to interpret and simply shows that an individual’s risk of an episodes increases with increasing malaria prevalence in their surrounding area. What panel C shows is that of similarly aged individuals, those with a higher number of previous episodes are at a higher risk of subsequent episodes. Panel D shows this the other way around, i.e. that of children with similar numbers of previous infections, those that are older are more protected. And this interaction between age and previous number of episodes is what explains the overall small effect size of age when not accounting for it (shown in Fig 7). We have now added a more detailed interpretation of these results to the main text. With regards to age itself, and plotting panel C twice, as shown in Fig 5, the effect of the previous number of episodes does not appear to decline within the age groups considered in this analysis. Furthermore, as the data is centred, the results implicitly compare young and old children. We do agree, however, that at some point in their lives this effect should diminish; unfortunately, though, we do not have the data to explore this further.  
- *Same query for panel D – for what number of previous episodes does this age effect apply?*
**Response:** Please see response to previous comment.
- *Can you comment on the strength of correlation between previous episodes and the prevalence index? Is it possible to distinguish the two effects from each other during the regression? E.g. can a child with say 20 previous episodes come from a lower PI area?*
**Response:** This is unfortunately not possible for this regression analysis simply because the prevalence index varies so much both over time and space, meaning it is not possible to categorise children based on PI in a meaningful way for more than a year or two. Furthermore, we have mapped the cohort by number of previous episodes (results not shown) and could not find any clear spatial clustering of individuals with high a low number of episodes. And although we would expect that children with many episodes must come from areas where there was a lot of transmission, it is possible to find other children living in the same area with much fewer recorded episodes, which to us suggests substantial individual-level variation in the risk of experiencing a malaria episode. 
- *A very interesting discussion. Could you explain this sentence in the last paragraph some more? “children experience different transmission intensities at different times during their life” – I was not sure how this was inferred.*
**Response:** This statement is based on the fact that malaria prevalence in this cohort varies significantly across space and time, as illustrated in Fig. 2. So for example, a child living in the South-West might have experienced high malaria transmission during early childhood and much less so in later years, whereas a child growing up in the North-East would have been exposed to less malaria during the first few years of their life but more so at a later stage during their childhood. We currently do not know if or how this affects an individual’s ability to acquire protective immunity, but maybe this is a first step in acknowledging that these differences exist and can arise even within a small geographic area.   
